# Supplementary material for: Glucose promotes cell growth by suppressing branched-chain amino acid degradation
Source: Nat Commun. 2018 Jul 26;9:2935. doi: 10.1038/s41467-018-05362-7 (PMC6062555; doi:10.1038/s41467-018-05362-7)
Supplement: Supplementary file 1 — Supplementary Information [file 41467_2018_5362_MOESM1_ESM.pdf]

Supplementary Information

Glucose Promotes Cell Growth by Suppressing Branched-chain Amino Acid Degradation

shao et al.

Supplementary Figure 1

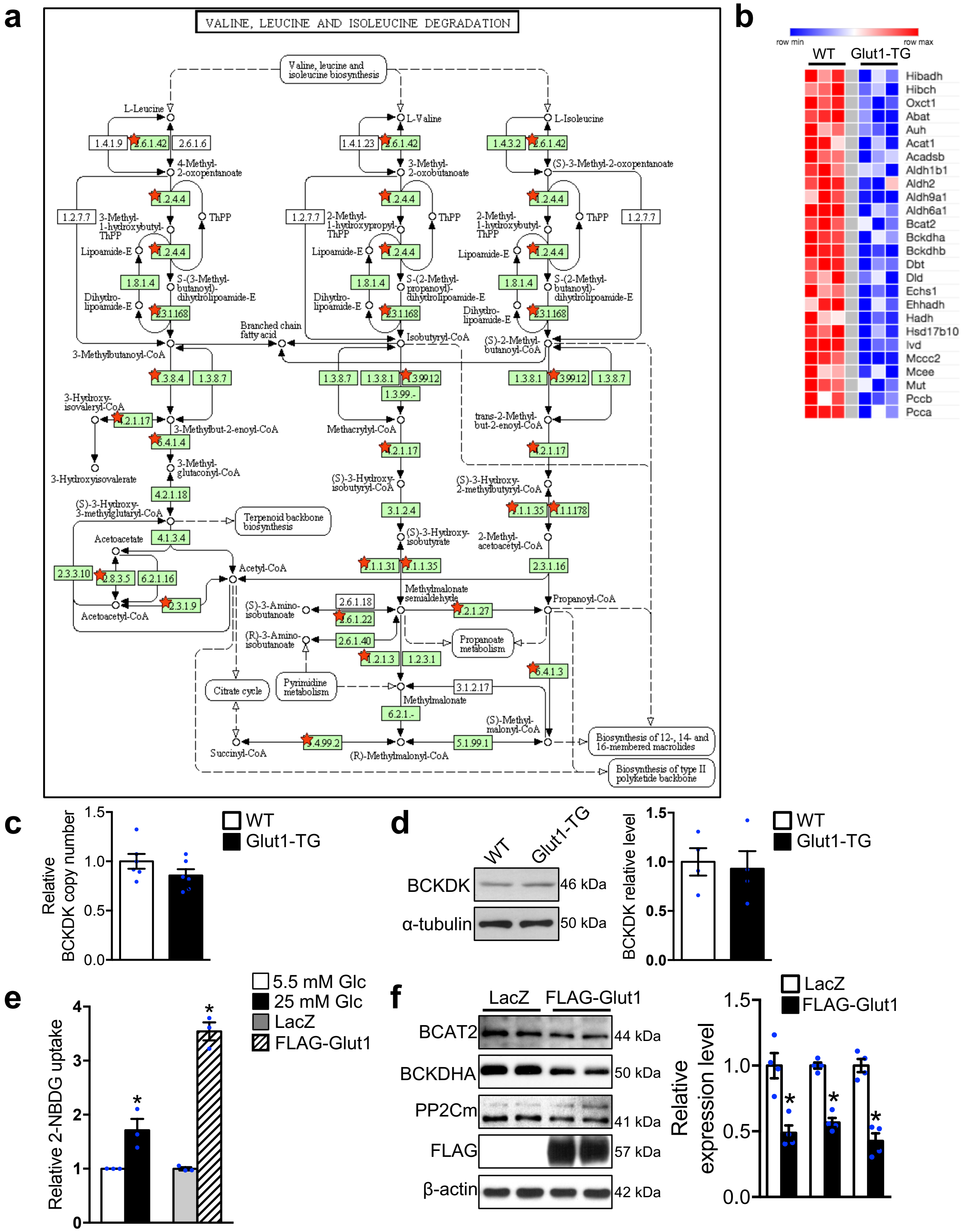

Supplementary Figure 1, continued

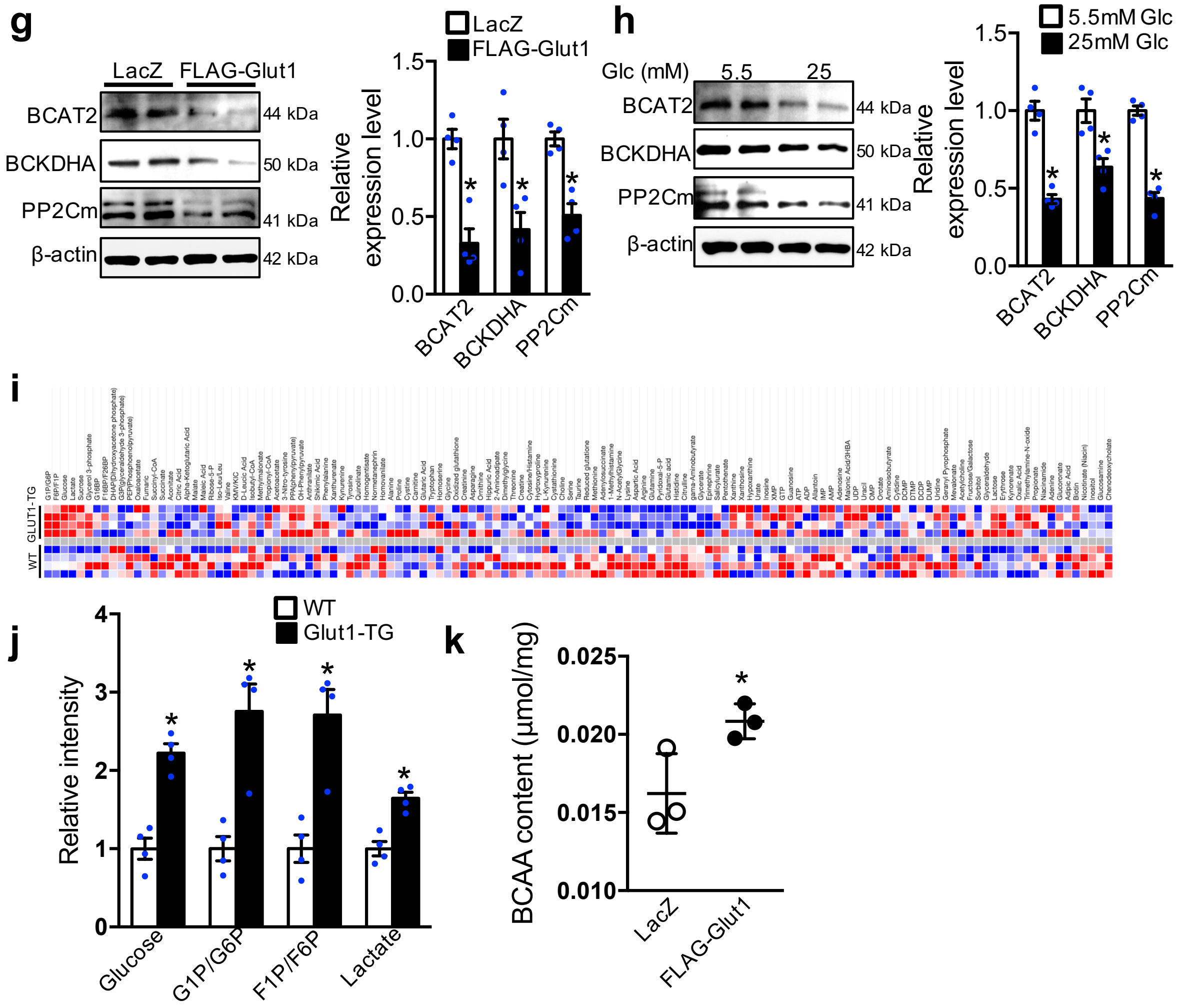

Supplementary Figure 1

(**a**) The downregulated genes in Glut1-TG hearts were identified and mapped onto the BCAA degradation pathway by KEGG (Kyoto Encyclopedia of Genes and Genomes). (**b**) Heat map of the expression of genes involved in BCAA degradation in Glut1-TG and WT hearts. Color coding for each gene was assigned using a log2 fold change versus the mean value of WT (n=3). (**c**) qRT-PCR analysis of BCKDK in Glut1-TG and WT mouse hearts. The expression was normalized to 18S rRNA and reported as fold change over WT (n=6). (**d**) Representative immunoblots of BCKDK and α-tubulin in heart tissue homogenates (left) and the densitomeric measurement of BCKDK (right) are shown (n=4). (**e**) NRCMs were incubated with DMEM containing 5.5 mM or 25 mM glucose or transfected with indicated adenovirus for 24 hours. 2-NBDG uptake was measured and relative fluorescent intensity was reported as fold change to the control (*p<0.05 vs. 5.5 mM Glc or LacZ, n=3). 2-NBDG, 2-deoxy-2-[(7-nitro-2,1,3-

benzoxadiazol-4-yl)amino]-D-glucose. (**f**-**h**) Immunoblots of cell lysates from NRCMs (**f**) or H9C2 cells (**g**, **h**) incubated with DMEM containing 5.5 mM or 25 mM glucose or transfected with indicated adenovirus for 24 hours (left) and statistical analyses of densitomeric measurements of BCAT2, BCKDHA and PP2Cm (right) are shown (*p<0.05 vs. LacZ or 5.5 mM Glc, n=4). (**i**) Heat map of metabolites measured by targeted metabolomics of Glut1-TG and WT heart tissues. Color coding for each metabolite was assigned using a log2 fold change versus the mean value of WT (n=4). (**j**) The relative intensity of indicated glycolytic metabolites measured by targeted metabolomics of Glut1-TG and WT mouse hearts (*p<0.05 vs. WT, n=4). G1P, Glucose-1-phosphate; G6P, Glucose-6-phosphate; F1P, Fructose-1-phosphate; F6P, Fructose-6-Phosphate. (**k**) Cellular BCAA levels in NRCMs transfected with indicated adenovirus for 24 hours (*p<0.05 vs. LacZ, n=3). Data shown as mean±s.e.m. *P* values were determined using unpaired Student's t-test (**e, f, g, h, j, k**) or Mann-Whitney test (**e, g, j**).

Supplementary Figure 2

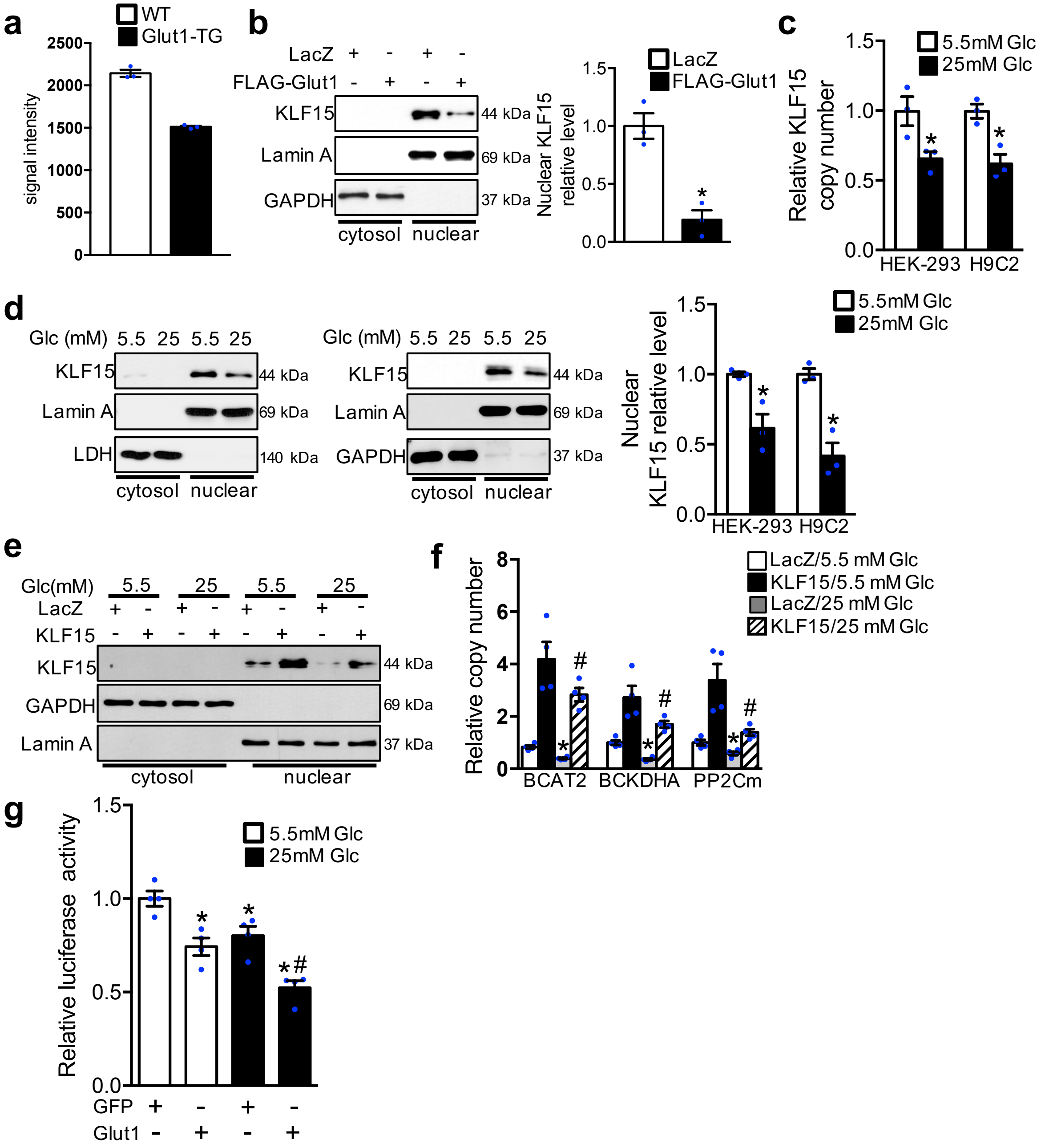

Supplementary Figure 2

(**a**) KLF15 gene expression in WT and Glut1-TG mouse hearts based upon microarray analysis (n=3). (**b-d**) Cells were incubated with DMEM containing 5.5 mM or 25 mM glucose or transfected with indicated adenovirus for 24 hours. (**b**) Immunoblots of cytosolic and nuclear fraction from NRCMs (left) and statistical analysis of densitomeric measurement of nuclear KLF15 (right) are shown (*p<0.05 vs. LacZ, n=3). (**c**) mRNA levels of KLF15 normalized to 18S rRNA (*p<0.05 vs. 5.5 mM Glc, n=3). (**d**) Immunoblots of cytosolic and nuclear fractions from HEK-293 cells (left), H9C2 cells (middle) and statistical analysis of densitomeric measurement

of nuclear KLF15 (right) are shown (*p<0.05 vs. 5.5 mM Glc, n=3). (**e-f**) NRCMs transduced with indicated adenovirus were incubated with DMEM containing 5.5 mM or 25 mM glucose for 24 hours. (**e**) Representative immunoblots of KLF15, GAPDH and Lamin A are shown. (**f**) qRT-PCR analysis of BCAT2, BCKDHA and PP2Cm. The expression was normalized to 18S rRNA and reported as fold change over the control (*p<0.05 vs. LacZ/5.5 mM Glc, #p<0.05 vs. LacZ/25 mM Glc, n=4). (**g**) NRCMs were co-transfected with KLF15 promoter luciferase reporter gene (-1068-luc) and either control (GFP) or Glut1 plasmid. Six hours after transfection cells were incubated with DMEM containing 5.5 mM or 25 mM glucose for 36 hours. The luciferase activity was measured (*p<0.05 vs. GFP/5.5 mM Glc, #p<0.05 vs. GFP/25 mM Glc, n=4). Data shown as mean±s.e.m. *P* values were determined using unpaired Student's t-test (**b, c, d**) or one-way ANOVA followed by Newman-Keuls comparison test (**f, g**).

Supplementary Figure 3

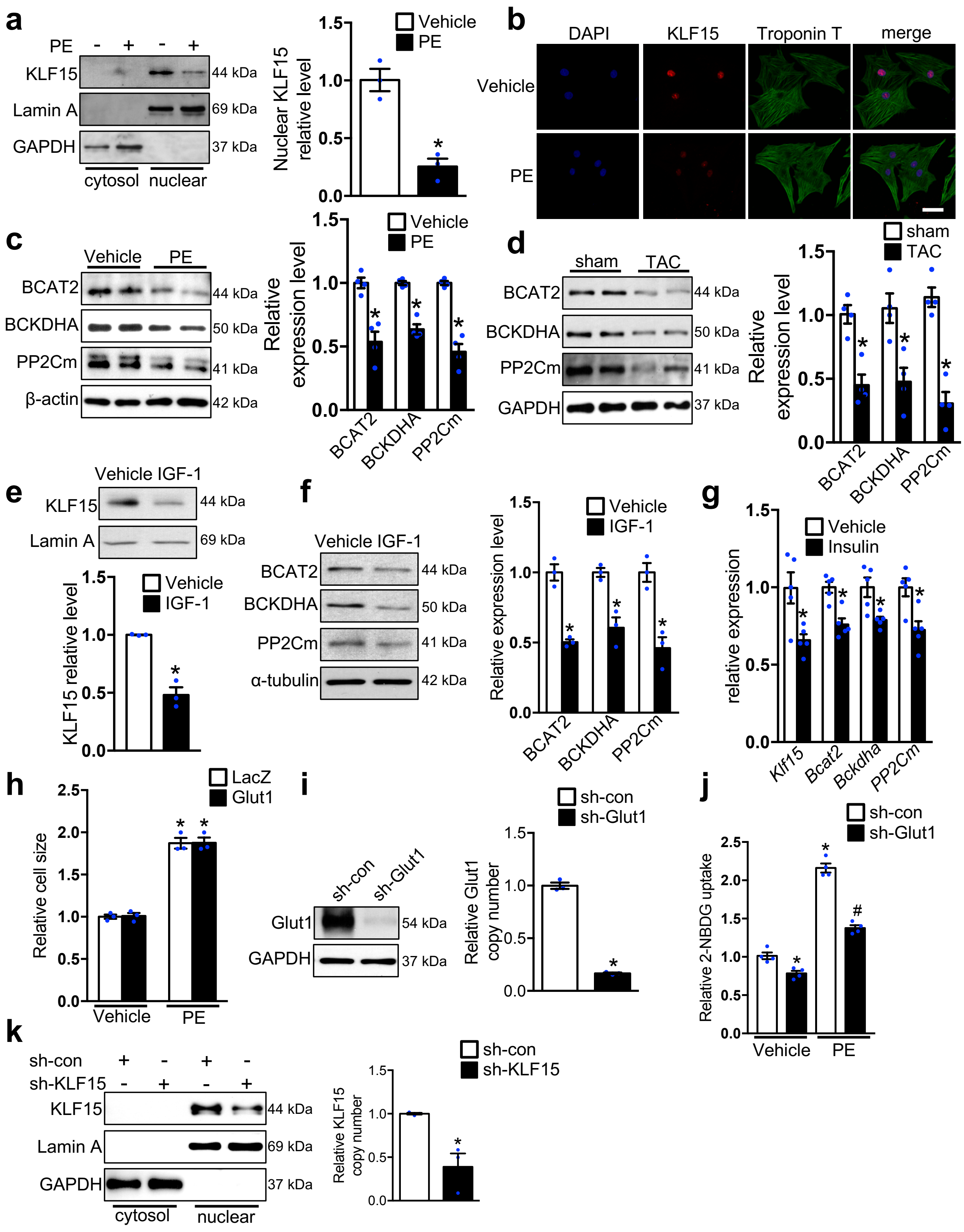

Supplementary Figure 3, continued

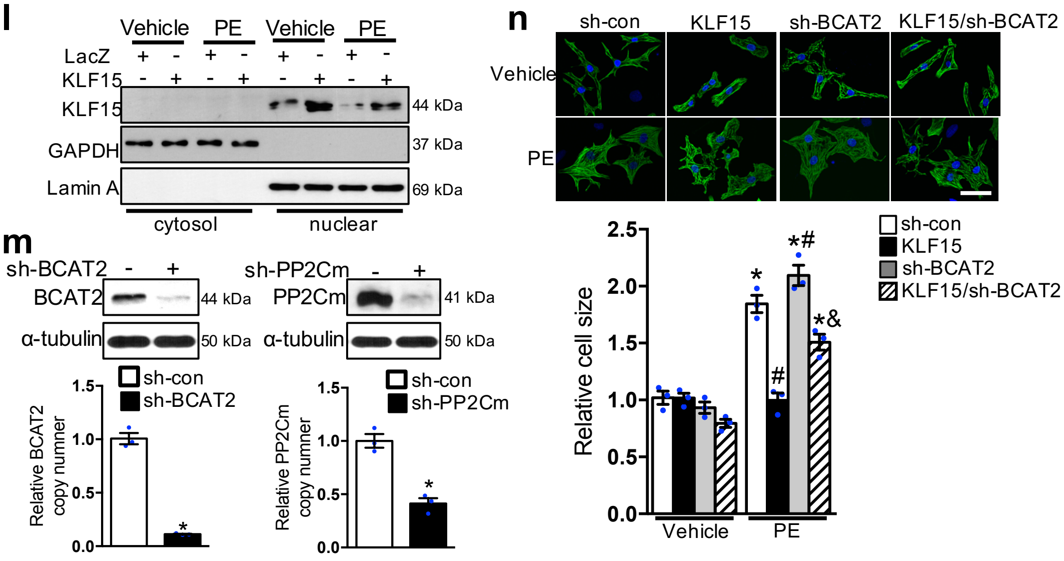

Supplementary Figure 3

(**a**) Immunoblots of cytosolic and nuclear fractions from NRCMs treated with phenylephrine (PE, 100 µM) or vehicle (left) and statistical analysis of densitomeric measurement of nuclear KLF15 (right) are shown (*p<0.05 vs. vehicle, n=3). (**b**) NRCMs were stained with a KLF15 antibody (red), a Troponin T antibody (green) and DAPI (blue). Scale bar, 25 µm. (**c**) Immunoblots of cell lysates from NRCMs treated with phenylephrine (PE, 100 µM) or vehicle (left) and statistical analyses of densitomeric measurements of BCAT2, BCKDHA and PP2Cm (right) are shown (*p<0.05 vs. Vehicle, n=4). (**d**) Immunoblots of heart homogenates from WT mice after TAC or sham surgery (left) and statistical analyses of densitomeric measurements of BCAT2, BCKDHA and PP2Cm (right) are shown (*p<0.05 vs. sham, n=4). (**e**) Immunoblots of nuclear fractions from NRCMs treated with insulin like growth factor 1 (IGF-1, 10 nM) or vehicle (upper) and statistical analysis of densitomeric measurement of nuclear KLF15 (lower) are shown (*p<0.05 vs. Vehicle, n=3). (**f**) Immunoblots of cell lysates from NRCMs treated with IGF1 (10 nM) or vehicle (left) and statistical analyses of densitomeric measurements of BCAT2, BCKDHA and PP2Cm (right) are shown (*p<0.05 vs. Vehicle, n=3). (**g**) WT mice were subjected to 18 hour fasting and then injected with vehicle or insulin (1 Unit per kg). Heart tissues were collected 2 hours after injection. The expressions of indicated genes were examined by qRT-PCR. The expression was normalized to 18S rRNA and reported as fold change over vehicle (*p<0.05 vs. Vehicle, n=5). (**h**) NRCMs transduced with indicated adenovirus were treated with phenylephrine (PE, 100 µM) or vehicle for 48 hours. Myocytes were fixed and stained with anti-Troponin T. Cell surface area in each group was quantified and expressed relative to the control (*p<0.05 vs. LacZ/Vehicle, n=3). (**i**) Glut1 protein and mRNA levels in NRCMs 72 hours after the transduction of indicated adenovirus (*p<0.05 vs. sh-con, n=3). (**j**) NRCMs transduced with indicated adenovirus for 72 hours were treated with phenylephrine (PE, 100 µM) or vehicle. 2-NBDG uptake was measured and relative fluorescent intensity was reported as fold change to the control. (*p<0.05 vs. sh-con/Vehicle, #p<0.05 vs. sh-con/PE, n=4). (**k**) KLF15 protein (left) and mRNA (right) levels in NRCMs 72 hours after the transduction of indicated adenovirus (*p<0.05 vs. sh-con, n=3). (**l**) NRCMs transduced with indicated adenovirus for 48 hours were treated with phenylephrine (PE, 100 µM) or vehicle. Representative immunoblots of KLF15, GAPDH and Lamin A are shown. (**m**) BCAT2 and PP2Cm protein and mRNA levels in NRCMs

72 hours after the transduction of indicated adenovirus (*p<0.05 vs. sh-con, n=3). (**n**) NRCMs transduced with indicated adenovirus were treated with phenylephrine (PE, 100 μM) or vehicle for 48 hours. Myocytes were fixed and stained with anti-Troponin T. Cell surface area in each group was quantified and expressed relative to the control (*p<0.05 vs. sh-con/Vehicle, #p<0.05 vs. sh-con/PE, &p<0.05 vs. KLF15/PE, n=3). Scale bar, 25 μm. Data shown as mean±s.e.m. *P* values were determined using unpaired Student's t-test (**a, c, d, e, f, g, i, k, m**), Mann-Whitney test (**d**), one-way ANOVA followed by Newman-Keuls comparison test (**j**) or Kruskal-Wallis test followed by Dunn's comparison test (**h, n**).

Supplementary Figure 4

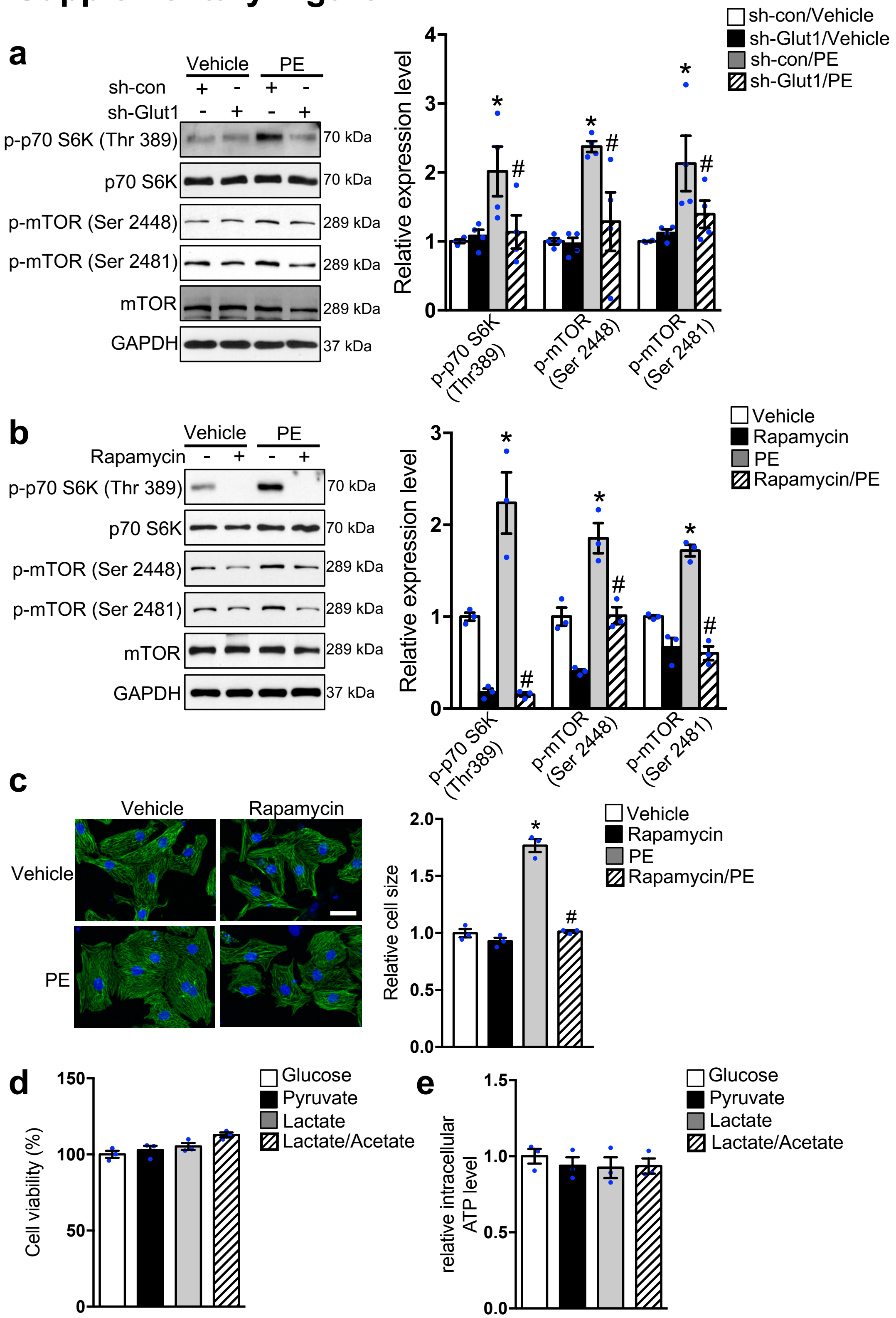

Supplementary Figure 4, continued

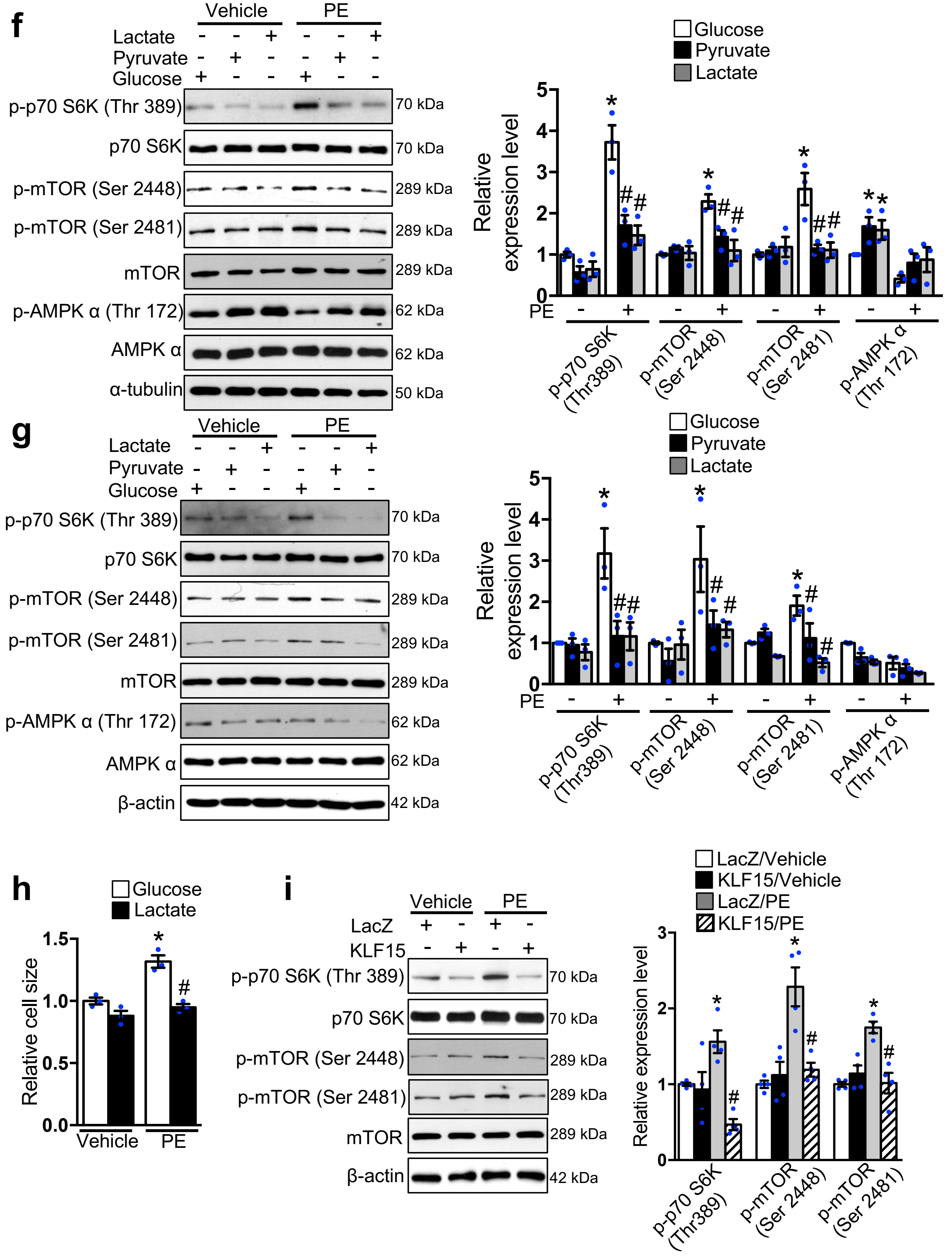

Supplementary Figure 4, continued

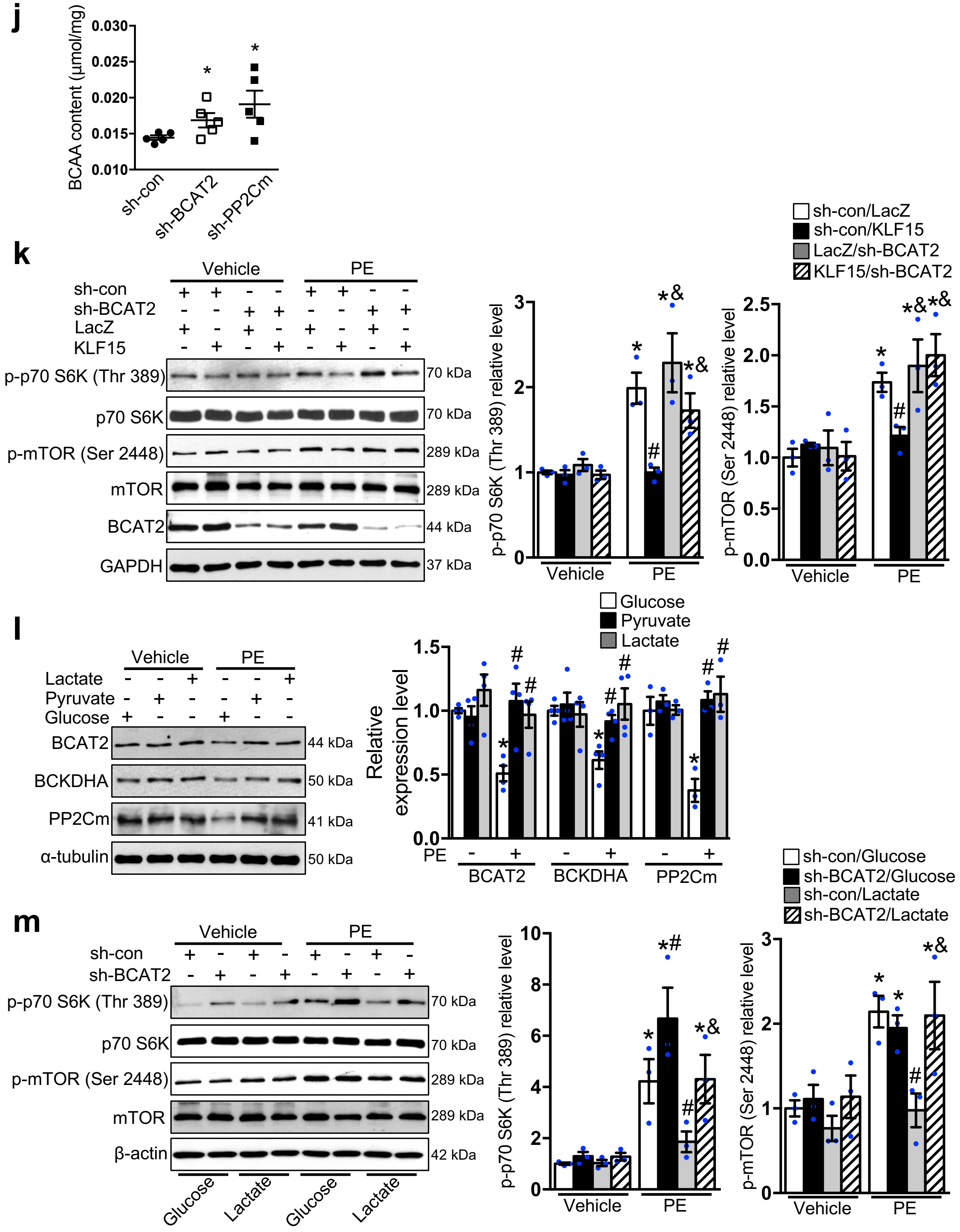

Supplementary Figure 4, continued

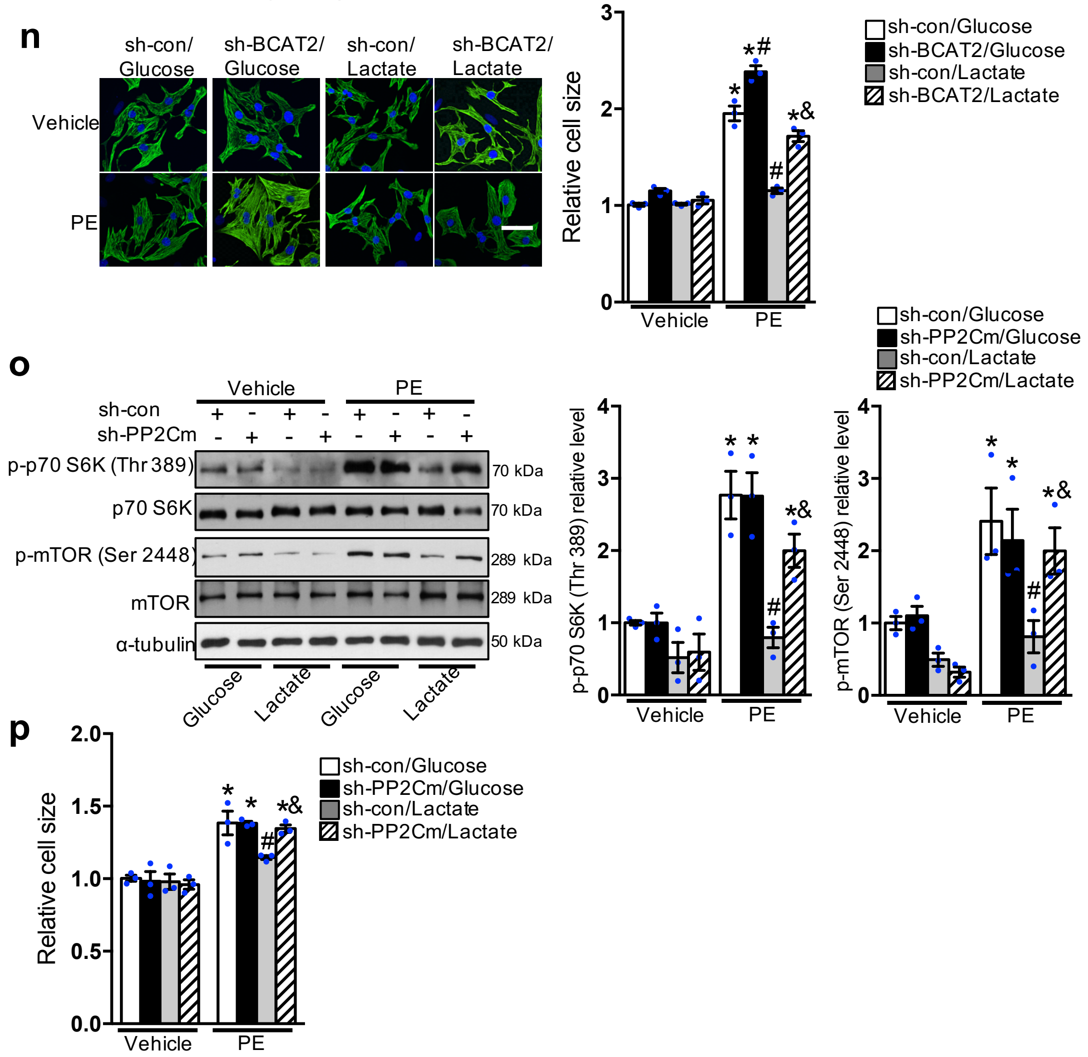

Supplementary Figure 4

(**a**) NRCMs transduced with indicated adenovirus were treated with phenylephrine (PE, 100 μM) or vehicle. Immunoblots of cell lysates (left) and statistical analyses of densitomeric measurements of p-p70 S6K (Thr 389), p-mTOR (Ser 2448) and p-mTOR (Ser 2481) (right) are shown (*p<0.05 vs. sh-con/Vehicle, #p<0.05 vs. sh-con/PE, n=4). (**b-c**) NRCMs with preincubation of rapamycin (100nM, 1 hour) or vehicle were treated with phenylephrine (PE, 100 μM) or vehicle. (**b**) Immunoblots of total cell lysates (left) and statistical analyses of densitomeric measurements of p-p70 S6K (Thr 389), p-mTOR (Ser 2448) and p-mTOR (Ser 2481) (right) are shown (*p<0.05 vs. Vehicle, #p<0.05 vs. PE, n=3). (**c**) Myocytes were fixed and stained with anti-Troponin T. Cell surface area in each group was quantified and expressed relative to the control (*p<0.05 vs. Vehicle, #p<0.05 vs. PE, n=3). Scale bar, 25 μm. (**d-e**) NRCMs were incubated with DMEM containing either glucose or non-glucose substrates for 48 hours. (**d**) Cell viability was measured (n=3). (**e**) Intracellular ATP content was measured (n=3). (**f**) NRCMs were pretreated with indicated medium for 1 hour and then incubated with phenylephrine (PE, 100 μM) or vehicle. Immunoblots of cell lysates (left) and statistical analyses of densitomeric measurements of p-p70 S6K (Thr 389), p-mTOR (Ser 2448), p-mTOR (Ser

2481) and p-AMPK α (Thr 172) (right) are shown (*p<0.05 vs. Glucose/PE(-), #p<0.05 vs. Glucose/PE(+), n=4). (**g**-**h**) Adult rat cardiomyocytes were pretreated with indicated medium for 1 hour and then incubated with phenylephrine (PE, 100 μM) or vehicle. (**g**) Immunoblots of cell lysates (left) and statistical analyses of densitomeric measurements of p-p70 S6K (Thr 389), p-mTOR (Ser 2448), p-mTOR (Ser 2481) and p-AMPK α (Thr 172) (right) are shown (*p<0.05 vs. Glucose/PE(-), #p<0.05 vs. Glucose/PE(+), n=3). (**h**) 48 hours after PE or vehicle treatment, cell size in each group was quantified and expressed relative to the control (*p<0.05 vs. Glucose/Vehicle, #p<0.05 vs. Glucose/PE, n=3). (**i**) NRCMs transduced with indicated adenovirus were treated with phenylephrine (PE, 100 μM) or vehicle. Immunoblots of cell lysates (left) and statistical analyses of densitomeric measurements of p-p70 S6K (Thr 389), p-mTOR (Ser 2448) and p-mTOR (Ser 2481) (right) are shown (*p<0.05 vs. LacZ/Vehicle, #p<0.05 vs. LacZ/PE, n=4). (**j**) BCAA levels in NRCMs transduced with indicated adenovirus for 72 hours (p<0.05 vs. sh-con, n=5). (**k**) NRCMs transduced with indicated adenovirus were treated with phenylephrine (PE, 100 μM) or vehicle. Immunoblots of cell lysates (left) and statistical analyses of densitomeric measurements of p-p70 S6K (Thr 389) and p-mTOR (Ser 2448) (right) are shown (*p<0.05 vs. sh-con/LacZ/Vehicle, #p<0.05 vs. sh-con/LacZ/PE, &p<0.05 vs. sh-con/KLF15/PE, n=3). (**l**) NRCMs were pretreated with indicated medium for 1 hour and then incubated with phenylephrine (PE, 100 μM) or vehicle. Immunoblots of cell lysates (left) and statistical analyses of densitomeric measurements of BCAT2, BCKDHA and PP2Cm (right) are shown (*p<0.05 vs. Glucose/PE(-), #p<0.05 vs. Glucose/PE(+), n=3-4). (**m**-**p**) NRCMs (**m**-**n**) or adult rat cardiomyocytes (**o**-**p**) transduced with indicated adenovirus were pretreated with indicated medium for 1 hour and then incubated with phenylephrine (PE, 100 μM) or vehicle. (**m** and **o**) Immunoblots of cell lysates (left) and statistical analyses of densitomeric measurements of p-p70 S6K (Thr 389) and p-mTOR (Ser 2448) (right) are shown (*p<0.05 vs. sh-con/Glucose/Vehicle, #p<0.05 vs. sh-con/Glucose/PE, &p<0.05 vs. sh-con/Lactate/PE, n=3). (**n** and **p**) 48 hours after PE or vehicle treatment, NRCMs (**n**) were fixed and stained with anti-Troponin T. Cell surface area in each group was quantified and expressed relative to the control (*p<0.05 vs. sh-con/Glucose/Vehicle, #p<0.05 vs. sh-con/Glucose/PE, &p<0.05 vs. sh-con/Lactate/PE, n=3). Scale bar, 25 μm. (**p**) Cell size in each group was quantified and expressed relative to the control (*p<0.05 vs. sh-con/Glucose/Vehicle, #p<0.05 vs. sh-con/Glucose/PE, &p<0.05 vs. sh-con/Lactate/PE, n=3). Data shown as mean±s.e.m. *P* values were determined using unpaired Student's t-test (**j**), one-way ANOVA followed by Newman-Keuls comparison test (**a**, **b**, **c**, **f**, **g**, **h**, **i**, **k**, **l**, **m, o, p**) or Kruskal-Wallis test followed by Dunn's comparison test (**a**, **l**, **m**, **n**).

Supplementary Figure 5

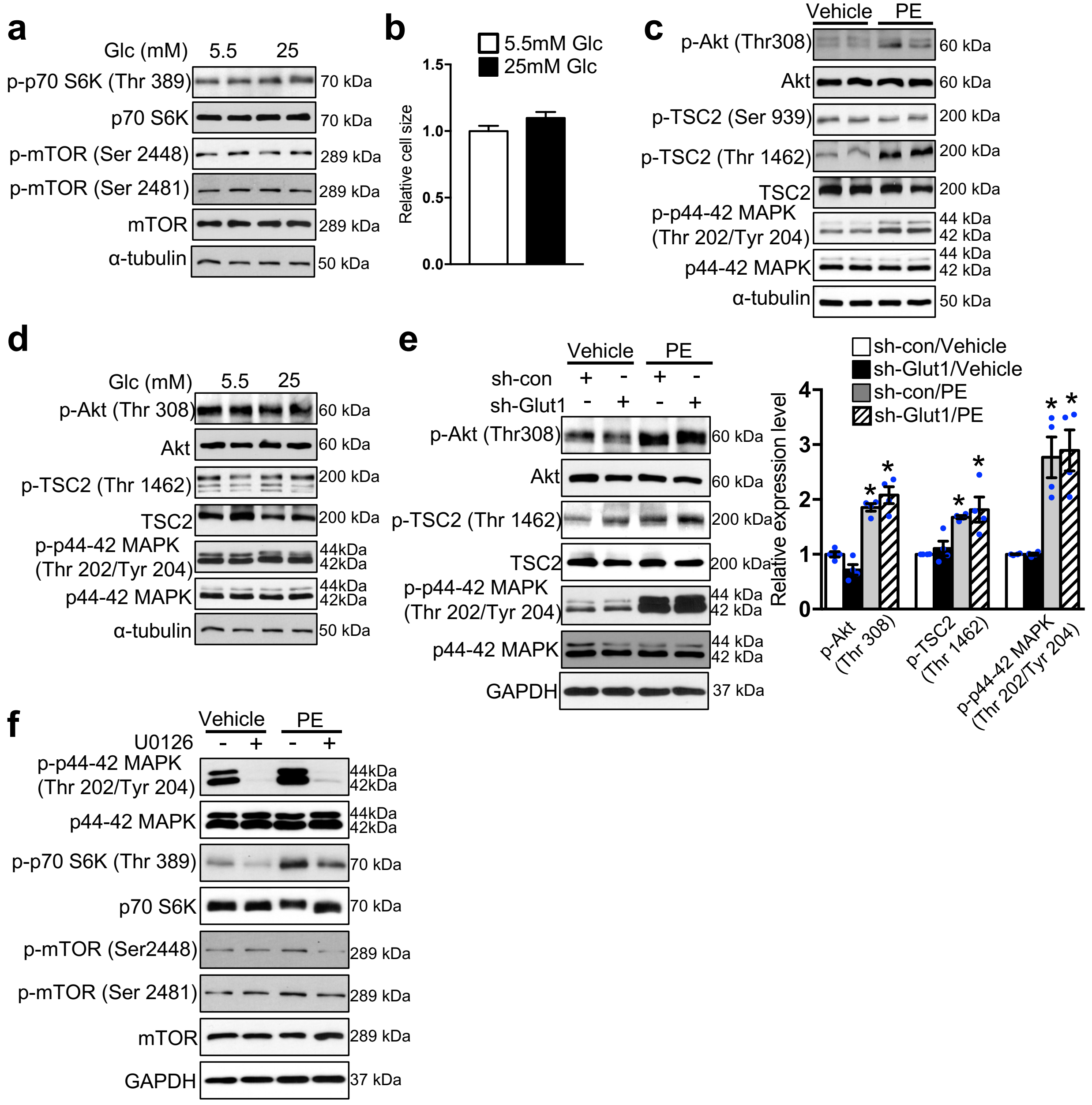

Supplementary Figure 5, continued

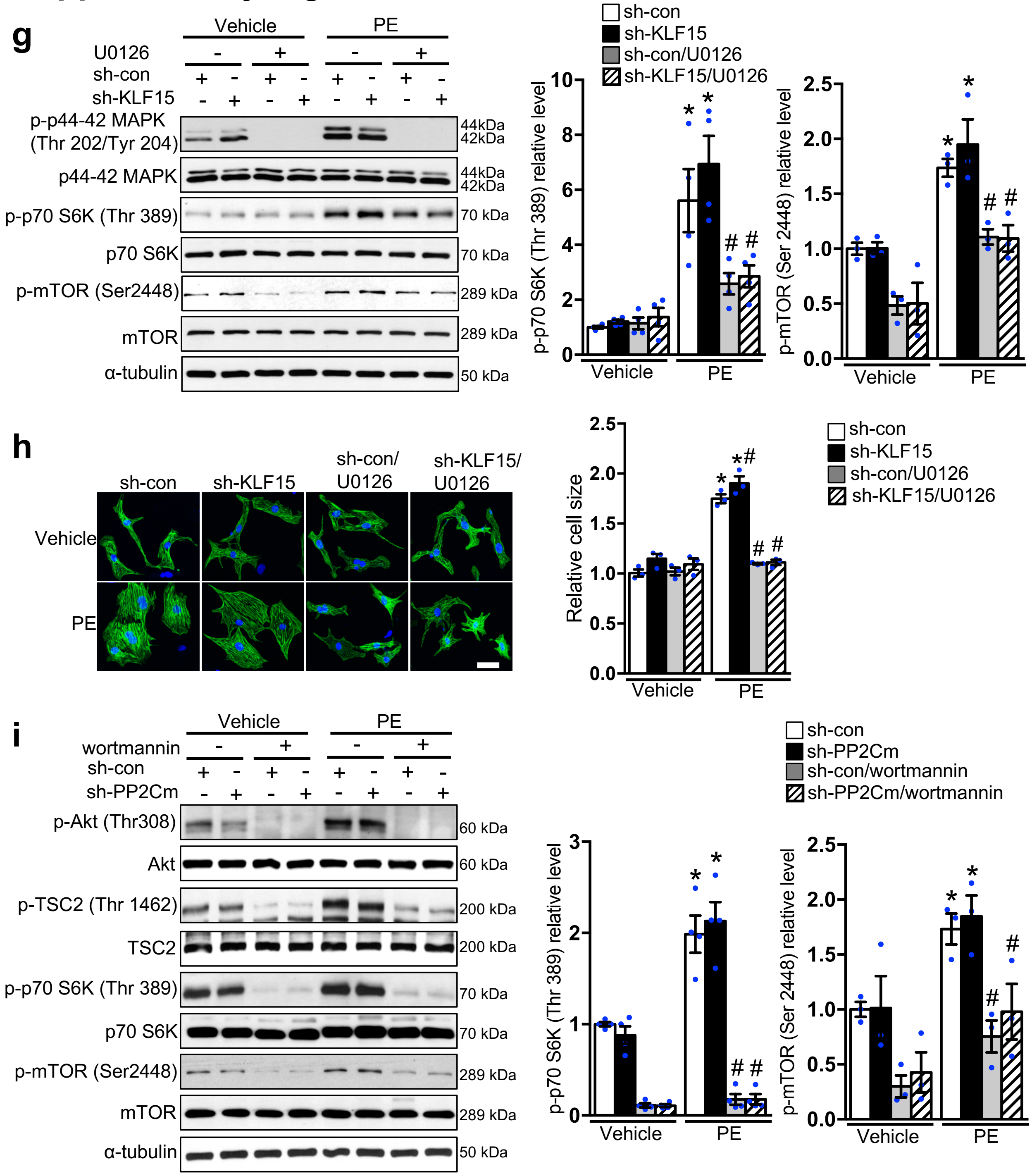

Supplementary Figure 5, continued

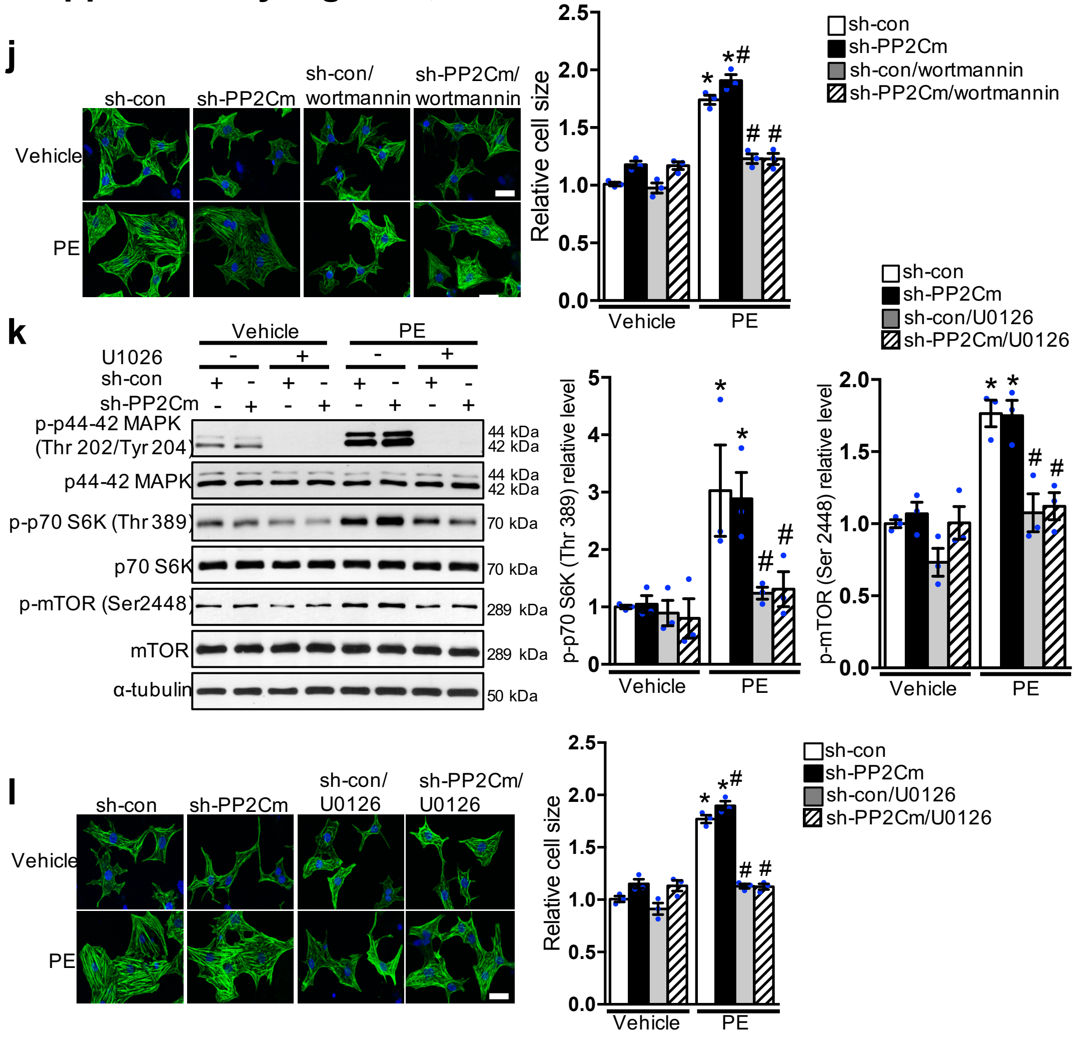

Supplementary Figure 5

(**a**) Immunoblots of cell lysates from NRCMs cultured with DMEM containing 5.5 mM or 25 mM glucose. (**b**) NRCMs were incubated with DMEM containing 5.5 mM or 25 mM glucose for 48 hours. Cell surface area in each group was quantified and expressed relative to the control (n=3). (**c**) Immunoblots of cell lysates from NRCMs treated with phenylephrine (PE, 100 μM) or vehicle. (**d**) Immunoblots of cell lysates from NRCMs cultured with DMEM containing 5.5 mM or 25 mM glucose. (**e**) NRCMs transduced with indicated adenovirus were treated with phenylephrine (PE, 100 μM) or vehicle. Immunoblots of cell lysates (left) and statistical analyses of densitomeric measurements of p-Akt (Thr 308), p-TSC2 (Thr 1462) and p-p44-42 MAPK (Thr 202/Tyr 204) (right) are shown (*p<0.05 vs. sh-con/Vehicle, n=4). (**f**) Immunoblots of cell lysates from NRCMs treated with phenylephrine (PE, 100 μM) or vehicle with or without preincubation of U0126 (10 μM, 1 hour). (**g**-**l**) NRCMs transduced with indicated adenovirus were preincubated with wortmannin (2 μM) or U0126 (10 μM) for 1 hour and subsequently treated with phenylephrine (PE, 100 μM) or vehicle. (**g**, **i** and **k**) Immunoblots of cell lysates (left) and statistical analyses of densitomeric measurements of p-p70 S6K (Thr 389) and p-mTOR (Ser 2448) (right) are shown (*p<0.05 vs. sh-con/Vehicle, #p<0.05 vs. sh-con/PE, n=3-4). (**h**, **j** and **l**) 48 hours after PE or vehicle treatment, myocytes were fixed and stained with anti-Troponin T.

Cell surface area in each group was quantified and expressed relative to the control (*p<0.05 vs. sh-con/Vehicle, #p<0.05 vs. sh-con/PE, n=3). Scale bar, 25 μm. Data shown as mean±s.e.m. *P* values were determined using one-way ANOVA followed by Newman-Keuls comparison test (**e**, **g**, **i**, **k**) or Kruskal-Wallis test followed by Dunn's comparison test (**h**, **j**, **l**).

Supplementary Figure 6

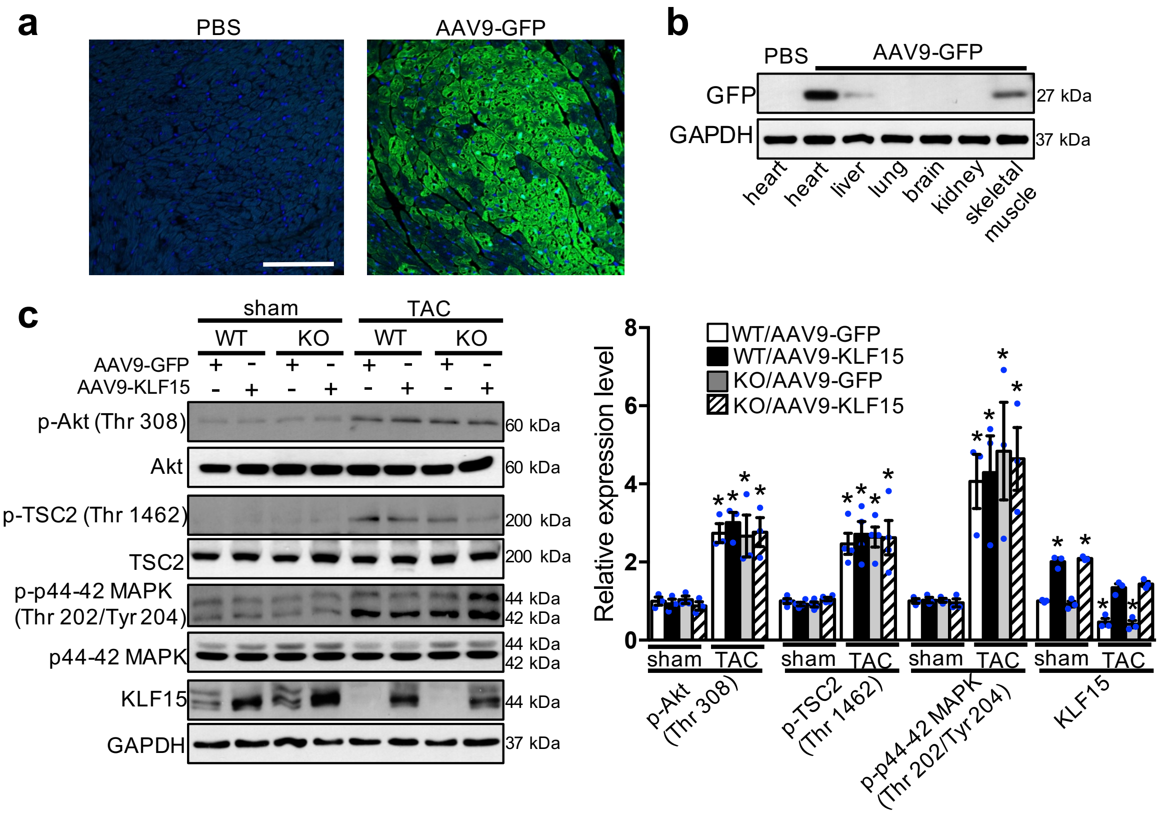

Supplementary Figure 6

(**a-b**) Tissue harvested one week after injection of AAV9-GFP or PBS. (**a**) Immunofluorescence detecting GFP (green) and DAPI (blue) in heart sections. Scale bar, 100 μm. (**b**) Immunoblots of tissue homogenates. (**c**) *PP2Cm* KO and WT mice were subjected to TAC or sham surgery one week after injection of AAV9-KLF15 or control virus (AAV9-GFP). Left ventricles from indicated hearts were collected 3 days post-surgery. Immunoblots of tissue homogenates (left) and statistical analyses of densitomeric measurements of p-Akt (Thr 308), p-TSC2 (Thr 1462), p-p44-42 MAPK (Thr 202/Tyr 204) and KLF15 (right) are shown (*p<0.05 vs. WT/AA9-GFP/sham, n=3-4). Data shown as mean±s.e.m. *P* values were determined using one-way ANOVA followed by Newman-Keuls comparison test (**c**).

Supplementary Figure 7

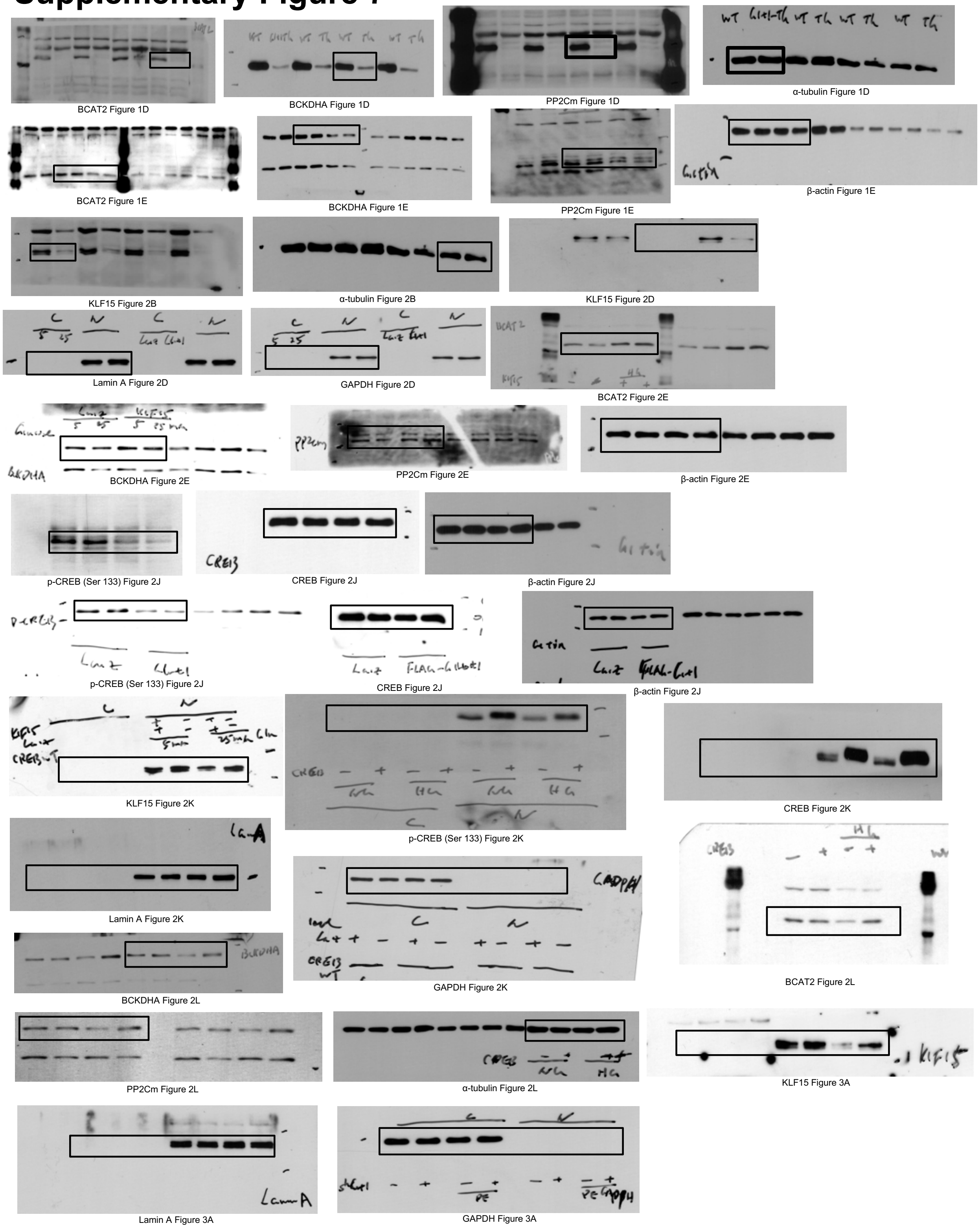

BCAT2 Figure 1D
BCKDHA Figure 1D
PP2Cm Figure 1D
α-tubulin Figure 1D
BCAT2 Figure 1E
BCKDHA Figure 1E
PP2Cm Figure 1E
β-actin Figure 1E
KLF15 Figure 2B
α-tubulin Figure 2B
KLF15 Figure 2D
Lamin A Figure 2D
GAPDH Figure 2D
BCAT2 Figure 2E
BCKDHA Figure 2E
PP2Cm Figure 2E
β-actin Figure 2E
p-CREB (Ser 133) Figure 2J
CREB Figure 2J
β-actin Figure 2J
p-CREB (Ser 133) Figure 2J
CREB Figure 2J
β-actin Figure 2J
KLF15 Figure 2K
p-CREB (Ser 133) Figure 2K
CREB Figure 2K
Lamin A Figure 2K
GAPDH Figure 2K
BCAT2 Figure 2L
BCKDHA Figure 2L
PP2Cm Figure 2L
α-tubulin Figure 2L
KLF15 Figure 3A
Lamin A Figure 3A
GAPDH Figure 3A

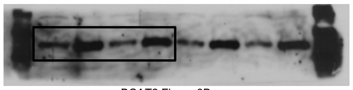
BCAT2 Figure 3B

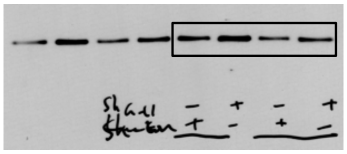
BCKDHA Figure 3B

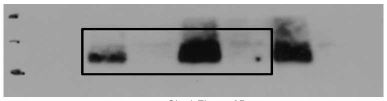
Glut1 Figure 3B

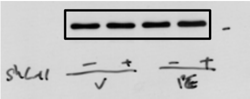
α-tubulin Figure 3B

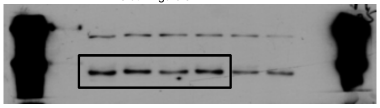
PP2Cm Figure 3E

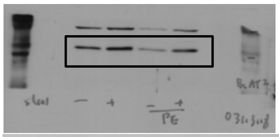
BCAT2 Figure 3E

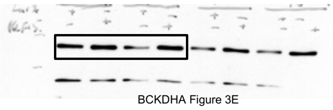
BCKDHA Figure 3E

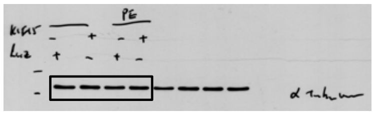
α-tubulin Figure 3E

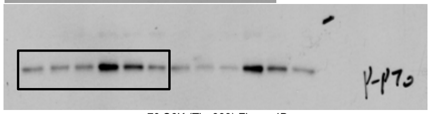
p-p70 S6K (Thr 389) Figure 4B

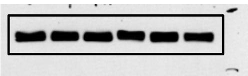
p70 S6K Figure 4B

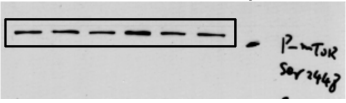
p-mTOR (Ser 2448) Figure 4B

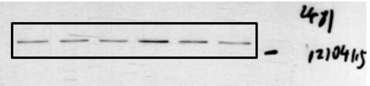
p-mTOR (Ser 2481) Figure 4B

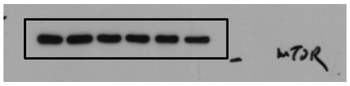
mTOR Figure 4B

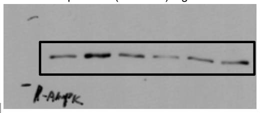
p-AMPK (Thr 172) Figure 4B

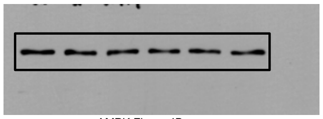
AMPK Figure 4B

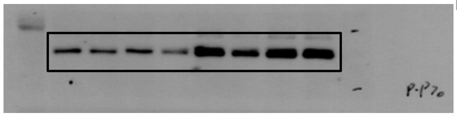
p-p70 S6K (Thr 389) Figure 4E

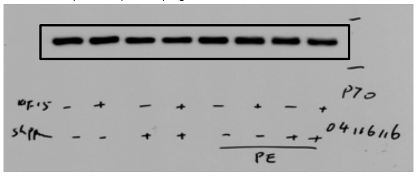
p70 S6K Figure 4E

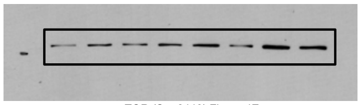
p-mTOR (Ser 2448) Figure 4E

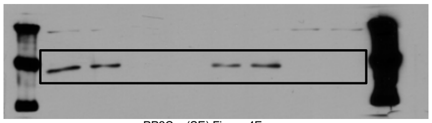
PP2Cm (SE) Figure 4E

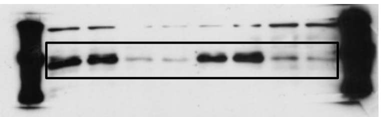
PP2Cm Figure 4E

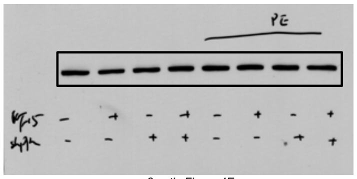
β-actin Figure 4E

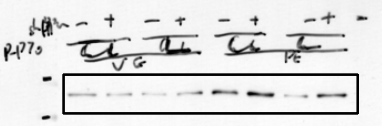
p-p70 S6K (Thr 389) Figure 4F

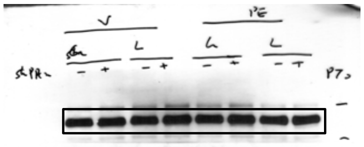
p70 S6K Figure 4F

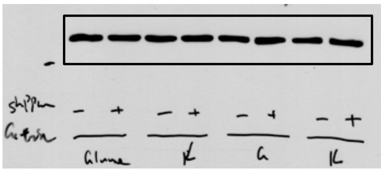
β-actin Figure 4F

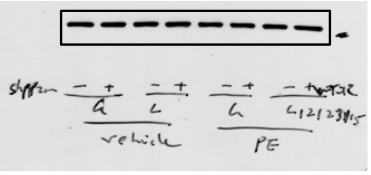
mTOR Figure 4F

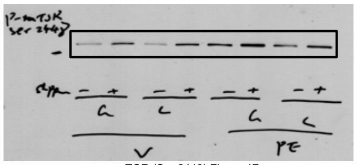
p-mTOR (Ser 2448) Figure 4F

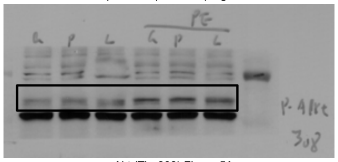
p-Akt (Thr 308) Figure 5A

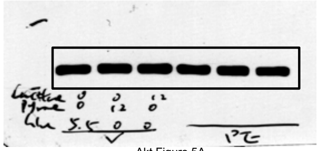
Akt Figure 5A

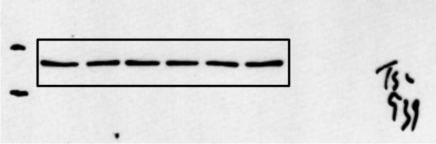
p-TSC2 (Ser 939) Figure 5A

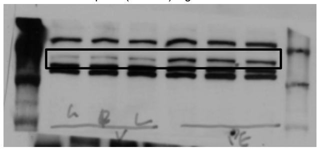
p-TSC2 (Thr 1462) Figure 5A

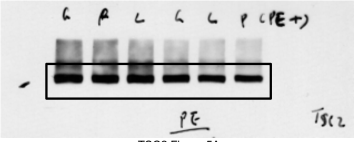
TSC2 Figure 5A

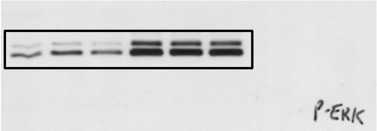
p-p44-42 MAPK (Thr 202/Tyr 204) Figure 5A

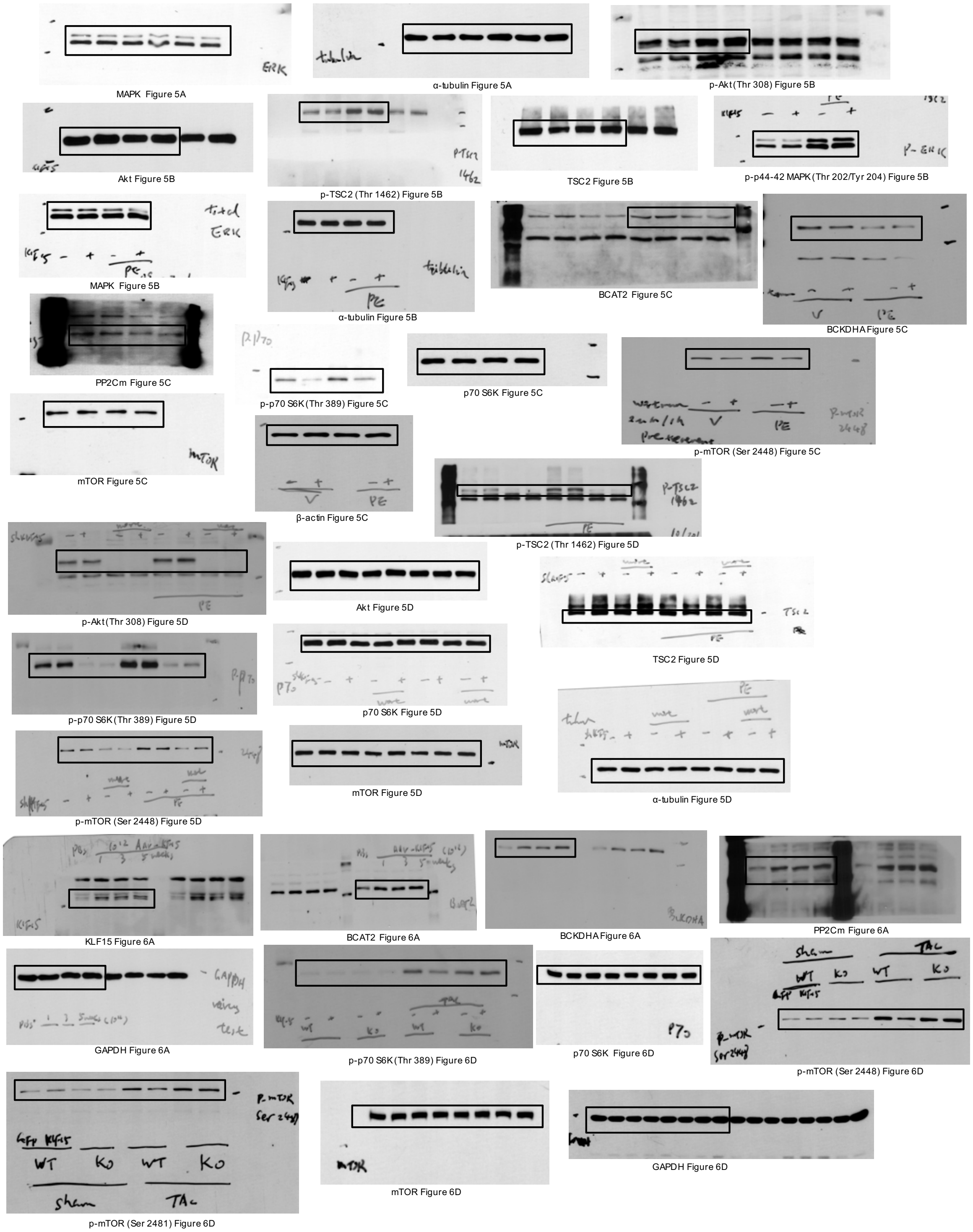

Supplementary Figure 7

Full blots for main text figures.

Supplementary Table 1

Term	Description	Count	P-Value	Fold Enrichment	Bonferroni	Benjamini	FDR
**Downregulated**							
mmu00280	Valine, leucine and isoleucine degradation	19	1.40E-14	11.3	3.60E-12	3.60E-12	1.80E-11
mmu01100	Metabolic pathways	77	1.10E-09	2	2.90E-07	1.50E-07	1.50E-06
mmu01130	Biosynthesis of antibiotics	24	1.20E-07	3.7	3.00E-05	1.00E-05	1.50E-04
mmu00640	Propanoate metabolism	8	1.20E-05	9.7	3.00E-03	7.50E-04	1.50E-02
mmu01200	Carbon metabolism	14	4.70E-05	3.9	1.20E-02	2.40E-03	6.10E-02
mmu00380	Tryptophan metabolism	9	7.40E-05	6.3	1.90E-02	3.10E-03	9.60E-02
mmu00071	Fatty acid degradation	9	1.00E-04	6	2.50E-02	3.70E-03	1.30E-01
mmu00310	Lysine degradation	8	9.20E-04	5	2.10E-01	2.90E-02	1.20E+00
mmu00650	Butanoate metabolism	6	1.20E-03	7.3	2.60E-01	3.30E-02	1.50E+00
mmu00410	beta-Alanine metabolism	6	3.00E-03	5.9	5.30E-01	7.30E-02	3.80E+00
mmu00480	Glutathione metabolism	7	6.20E-03	4.2	8.00E-01	1.40E-01	7.80E+00
mmu05204	Chemical carcinogenesis	9	6.80E-03	3.2	8.20E-01	1.30E-01	8.50E+00
mmu00500	Starch and sucrose metabolism	5	1.50E-02	5.1	9.80E-01	2.60E-01	1.80E+01
mmu00250	Alanine, aspartate and glutamate metabolism	5	2.50E-02	4.4	1.00E+00	3.70E-01	2.80E+01
mmu04530	Tight junction	10	2.60E-02	2.4	1.00E+00	3.60E-01	2.90E+01
mmu00620	Pyruvate metabolism	5	3.00E-02	4.2	1.00E+00	3.80E-01	3.30E+01
mmu00980	Metabolism of xenobiotics by cytochrome P450	6	4.50E-02	3.1	1.00E+00	5.00E-01	4.50E+01
mmu00982	Drug metabolism - cytochrome P450	6	5.00E-02	3	1.00E+00	5.20E-01	4.90E+01
**Upregulated**							
mmu04976	Bile secretion	9	7.10E-03	3.2	8.40E-01	8.40E-01	8.90E+00
mmu04530	Tight junction	12	2.30E-02	2.1	1.00E+00	7.80E-01	2.70E+01
mmu04660	T cell receptor signaling pathway	10	2.30E-02	2.4	1.00E+00	8.60E-01	2.60E+01
mmu04670	Leukocyte transendothelial migration	11	2.30E-02	2.3	1.00E+00	9.50E-01	2.60E+01
mmu04310	Wnt signaling pathway	12	2.60E-02	2.1	1.00E+00	7.40E-01	2.90E+01
mmu04070	Phosphatidylinositol signaling system	9	4.00E-02	2.3	1.00E+00	8.30E-01	4.10E+01

The top 24 KEGG pathways identified by DAVID in Glut1-TG hearts.

Supplementary Table 2

	sham				TAC 2 weeks				TAC 4 weeks			
	WT/AAV9-GFP	WT/AAV9-KLF15	KO/AAV9-GFP	KO/AAV9-KLF15	WT/AAV9-GFP	WT/AAV9-KLF15	KO/AAV9-GFP	KO/AAV9-KLF15	WT/AAV9-GFP	WT/AAV9-KLF15	KO/AAV9-GFP	KO/AAV9-KLF15
IVS;d (mm)	0.94±0.03	0.96±0.05	0.91±0.06	1.03±0.04	1.17±0.06	1.06±0.04	0.98±0.08	1.02±0.04	1.07±0.05	1.12±0.04	1.07±0.03	1.08±0.05
IVS;s (mm)	1.47±0.04	1.49±0.04	1.35±0.03	1.50±0.02	1.53±0.07	1.58±0.06	1.27±0.10#&	1.29±0.07&	1.41±0.09	1.58±0.11	1.35±0.05	1.32±0.06
LVID;d (mm)	3.24±0.12	3.17±0.11	3.22±0.27	3.25±0.06	3.86±0.14	3.52±0.27	3.92±0.32	3.94±0.20	4.22±0.24	3.81±0.292	4.15±0.21	4.13±0.23
LVID;s (mm)	1.85±0.08	1.92±0.07	1.86±0.15	1.95±0.07	3.14±0.18*	2.39±0.37#	3.33±0.30*&	3.40±0.24*&	3.65±0.30*	2.80±0.40	3.60±0.27*	3.65±0.27*
LVPW;d (mm)	0.81±0.04	0.78±0.04	0.94±0.07	0.84±0.06	1.04±0.06	1.03±0.04	0.90±0.07	0.99±0.05	1.05±0.05*	1.05±0.04*	0.94±0.05	0.99±0.05
LVPW;s (mm)	1.26±0.04	1.23±0.04	1.41±0.09	1.24±0.06	1.25±0.08	1.45±0.07	1.03±0.09&	1.14±0.06&	1.22±0.07	1.37±0.06	1.10±0.08	1.07±0.07
EF (%)	74.82±2.68	71.24±1.98	74.19±1.82	71.93±1.69	39.59±3.56*	62.28±7.35#	33.38±4.01*&	30.89±4.18*&	30.74±5.03*	53.66±7.64#	30.35±4.44*&	26.43±4.35*&
FS (%)	42.83±2.44	39.53±1.66	41.95±1.70	40.06±1.40	19.13±1.95*	34.17±4.79#	15.88±2.07*&	14.51±2.15*&	14.66±2.58*	28.35±4.72*#	14.45±2.33*&	12.32±2.23*&
LV Mass (mg)	95.23±3.35	91.65±6.16	101.84±10.04	105.22±6.28	174.02±6.61*	142.32±14.44*#	139.88±10.59*#	158.31±10.48*	187.80±9.89*	167.73±16.55*	169.09±8.64*	177.41±13.65*
LV Mass (Corrected, mg)	76.18±2.68	73.32±4.93	81.48±8.03	84.18±5.02	139.22±5.29*	113.86±11.55*#	111.90±8.47*#	126.64±8.38*	150.24±7.91*	134.18±13.24*	135.27±6.91*	141.93±32.75*
LV Vol;d (ml)	42.68±3.56	40.56±3.36	42.37±8.36	42.45±1.72	65.31±5.46	54.60±10.62	70.19±9.02	69.39±7.79	82.98±10.50	65.60±12.00	79.84±9.65	78.19±9.55
LV Vol;s (ml)	10.58±1.21	11.59±1.05	10.88±2.27	11.99±1.16	40.93±5.31	24.88±10.13*	49.82±8.64*	50.20±7.50*	61.43±10.96*	35.57±11.75	59.46±10.39*	60.16±9.46*
HR (bpm)	570±16	574±12	585±11	600±6	568±11	569±14	547±41	585±16	560±6	549±27	553±7	560±11

Echocardiography examination of *PP2Cm* KO mice and their control littermates with indicated AAV injections 2 and 4 weeks after TAC or sham surgery (*p<0.05 vs. WT/AA9-GFP/sham, #p<0.05 vs. WT/AAV9-GFP/TAC, &p<0.05 vs. WT/AAV9-KLF15/TAC, n=5-12). Data shown as mean±s.e.m. *P* values were determined using one-way ANOVA followed by Newman-Keuls comparison test.

Supplementary Table 3

Description	Species	Forward sequence	Reverse sequence
KLF15 promoter-CRE1	Rat	AGACTTCCTCAGTGCTCTGA	ATGCCAAGGACATTTGGACA
KLF15 promoter-CRE2	Rat	CAGTACAGGTCCACGAAGAG	CTCTCCCTGCTCTCCGAC
KLF15	Human	GTGAGAAGCCCTTCGCCT	CACACAGGACACTGGTACGG
18S	Human	ATCACCATTATGCAGAATCCACG	GACCTGGCTGTATTTTCCATCC
Bcat2	Mouse	CGGAACGAGCCTCTACGTG	GCTTGTGTGACCATACCAACA
Bckdha	Mouse	CTCCTGTTGGGACGATCTGG	CATTGGGCTGGATGAACTCAA
Bckdhb	Mouse	AGCTATTGCGGAAATCCAGTTT	ACAGTTGAAAAGATCACCTGAGC
Dbt	Mouse	TCTCTGGGACTCCTGCAGTT	GGACAATTAACCCCAGCTCA
PP2Cm	Mouse	AAACCCATGAAGCTGACCAC	GGCTGTCCCAAACTATTCCA
Ivd	Mouse	CATGGCAAGTCGACAGTACG	CTCGGCTGCATACAGAATCA
Mccc2	Mouse	GCCTATCACGGGGACTCAGT	CACTCCCTCCTAGTCTCACATAC
Mccc1	Mouse	GGGTGATACGAACAGCCAAAA	CTCGTCTGCCATATCTACATGC
Pccb	Mouse	GGACACCAAGATGTGGAAGC	TCCGAGCACGAGTAGAGGAT
Pcca	Mouse	AGCCAAGAGAGCAAAGGTCA	TCACAGGGTAGCCAATTTCC
Mut	Mouse	CGGCCAGATATCCTTGTCAT	GGACAGCAGCTCTTGGAATC
Bckdk	Mouse	ACCTATGCATGGCTTTGGCT	CGTAGGTAGACATCCGTGCC
KLF15	Mouse	CAGAGAGCGTCAAGGTCGC	TTCGCACAAACTTTGAGGGCA
Nppa	Mouse	GCTTCCAGGCCATATTGGAG	GGGGGCATGACCTCATCTT
Nppb	Mouse	AGTCCTTCGGTCTCAAGGCA	CCGATCCGGTCTATCTTGTGC
18S	Mouse	GGACAGGATTGACAGATTGATAG	ATCGCTCCACCAACTAAGAA
KLF15	Rat	AGACGTTGTGCTGCTTTCCT	CATTTCGGTGACGAGAAGGT
Glut1	Rat	GTGTGCAGCAGCCTGTGTAT	ACAGCGACACCACAGTGAAG
Bcat2	Rat	TCCAGAACCTCACACTGCAC	GAGGCGTACCTGCTTGTCTC
Bckdha	Rat	CAACGATGTGTTTGCGGTGT	TGCCCGATCCTGTAGGTCAT
PP2Cm	Rat	AGCCCTGTTGAGAGATGGTG	CACCGCATTTCTTGATCCTT
18S	Rat	GACAGGATTGACAGATTGATAGC	AGTCTCGTTCGTTATCGGAAT

A list of primer sequences used for quantitative RT-PCR measurements.
